# Awareness, attitudes and perceptions of students towards leisure noise in Durban, South Africa

**DOI:** 10.4102/sajcd.v71i1.1040

**Published:** 2024-06-28

**Authors:** Husna Mahomed, Seema Panday

**Affiliations:** 1 Discipline of Audiology, School of Health Sciences, University of KwaZulu-Natal, Durban, South Africa

**Keywords:** leisure noise, awareness, attitudes, perceptions, hearing conservation programmes, hearing protection devices, personal listening devices, young adult

## Abstract

**Background:**

Young adults are exposed to high noise levels in leisure venues, which increases their risk of hearing loss, and can affect their quality of life.

**Objectives:**

The aim of this study was to describe the young adults’ awareness, attitudes and perceptions towards leisure noise at a university in South Africa.

**Method:**

A descriptive cross-sectional study design with quantitative methods of data was considered for this study. Students from first to fourth years in the Education Department of a local university in Durban, South Africa, who were aged 18 years old – 25 years old were invited to participate in an online survey.

**Results:**

Of the 462 participants, most had a general awareness on noise and hearing loss but lacked knowledge on the negative effect of loud noise, with 95.2% using personal listening devices, followed by visiting restaurants and gyms, and 48.3% being unsure if noise can damage hearing permanently. They were unaware of methods to reduce their exposure to noise. A significant relationship between awareness of noise and attitudes (*p* = 0.029) indicated that the higher the level of awareness regarding leisure noise, the better their attitude and behaviour, thus the lower the risk of hearing loss.

**Conclusion:**

The results highlight the need for implementing the World Health Organization (WHO) noise regulations and providing education for this age group to prevent irreversible hearing loss through exposure to leisure noise.

**Contribution:**

A national study is recommended to increase research evidence.

## Introduction

Leisure noise is a common phenomenon at gyms, clubs and for listening through the use of personal listening devices (PLDs) (World Health Organization [WHO], 2018). While there is considerable evidence that exposure to loud noise over extended periods can be harmful to hearing, there is little public health education (WHO, 2015a, 2018, 2020) in South Africa (SA) about the causes and ways to prevent a noise-induced hearing loss (NIHL). Very few studies have been conducted in SA regarding individuals’ awareness, attitudes and perceptions regarding NIHL in relation to leisure, specifically among young adults who are exposed to leisure noise as part of their daily lives. Previous studies have focused more on occupational noise (Joubert et al., 2017; Kidane, 2015) and presbycusis (Lin et al., 2013). Approximately 33% of hearing loss is believed to be related to high noise levels (Almaayeh et al., 2018). Of the 466 million people globally who experience difficulty hearing, most are in Asia and Africa (WHO, 2018), of whom 93% are adults (WHO, 2019a). By 2030, an estimated 630 million people will be affected with hearing loss unless urgent action is taken (WHO, 2019a).

Noise is regarded as a sound that is loud or disturbing (South African Association of Audiologists, 2020). The two types of noise exposure explored in this study are recreational and occupational (in the place of employment, such as, factories and mines), both of which can have short- and long-term consequences. Occupational noise in the workplace is normally regulated by legislations, policies and practices to ensure better hearing health for the employees to minimise the occurrence of NIHL (SANS 10083, 2021). However, this is not the case for recreational noise, despite the exposure to risky listening behaviour, which is behaviour that increases the likelihood of a disease or injury (Tariq & Gupta, 2021), or loud noise levels for extended periods, causing potential harm (WHO, 2018). This can occur at entertainment venues, sporting events, gyms and excessive use of high-volume PLDs (WHO, 2018).

Hearing loss due to loud noise levels damages the structures or nerves in the inner ear that respond to sound (WHO, 2015b). Such sounds can cause irreversible severe damage to the inner ear (Punch et al., 2011). Noise exposure generally results in a sensorineural type of hearing loss, which is usually bilateral, and often presents as a notch on the audiogram at 4000Hz, with NIHL tending to affect higher frequencies first (Mathur, 2018). Loud music exposure is of particular concern among young music listeners, who are likely to experience some level of hearing loss by their mid-twenties (Carter et al., 2014; Sadhra et al., 2002). Approximately 15% of young adults (18 years – 25 years) are frequently exposed to recreational noise and are therefore at high risk of hearing loss due to their noise exposure (Carter et al., 2014).

An online survey of 993 young Australian participants aimed to establish if the sound levels of entertainment venues they frequent were regarded as acceptable. Results indicated that most respondents experienced hearing difficulties shortly after departing, with 75% noting that they preferred lower sound levels (Beach & Gilliver, 2019). According to the South African National Standards (SANS) (2021), sound levels over 85 dB for an extended period are considered detrimental to hearing (SANS 10083, 2021). Sound levels in nightclubs generally range from 82 dB to 106 dB, and from 85 dB to 105 dB at concerts (Beach et al., as cited by Beach & Gilliver, 2019).

Research indicates that 54% of the population use PLDs in developing countries and 87% in developed countries (WHO, 2018), half of whom listen to music in an unsafe manner (WHO, 2018). Approximately 50% use them at risky levels, and 5% – 10% could develop a NIHL due to the volume and duration of use (WHO, 2019b). Govindsamy et al. (2020) conducted a survey in KwaZulu-Natal (KZN) that investigated the views and behaviours of 107 undergraduate health science students towards the use of PLDs. Most (94%) increased the volume when in a noisy background, while 66% were aware that although NIHL was preventable, they continued to practise their risky behaviour. A further study on 269 health science students in SA to determine their knowledge on hearing loss and PLDs reported that 30% were unaware that PLDs increased the risk of a hearing loss (Seedat et al., 2020).

A study on noise levels at gyms revealed that the noise ranged from 93 dB to 101 dB during a spinning class (Shuster & Hertzano, 2021), this being considerably higher than the recommended levels for noise exposure of 85 dB (SANS 10083, 2021). Such high levels can result in hearing loss, both temporary and permanent, with temporary threshold shift (TTS) referring to a hearing loss as a shift in hearing threshold sensitivity that recovers back to its normal level in the hours and days following excessive noise exposure (Ryan et al., 2016). It is characterised by reduced sensitivity, feelings of fullness and tinnitus, with the degree of hearing loss and recovery time required being relative to the duration and intensity of noise (Ryan et al., 2016). A permanent threshold shift (PTS) refers to a hearing threshold shift that persists after the period of recovery post noise exposure and occurs when the threshold does not return to the pre-exposure level, resulting a NIHL (Ryan et al., 2016). The physiological changes that occur are that the swollen hair cells in the cochlea may rupture, become distorted and insufficiently transmit energy, which may also result in damage to the auditory nerve (Ryan et al., 2016).

Chung et al. (2005) evaluated NIHL in young people by using an online survey and hypothesised that individuals who lack awareness regarding the risks of noise are more likely to have a hearing loss. Sixty-one per cent of participants experienced tinnitus and 43% a TTS after exposure to loud music. Holmes et al. (2007) conducted a study on university students to estimate the prevalence of perceived hearing loss and TTS, as well as to establish if their attitude towards the noise affected their perception of these factors. Many people experienced TTS and pain with loud noise exposure, as well as perceived tinnitus and hearing loss. Their attitude towards daily loud noise was negative.

Gilles et al. (2012) conducted a study to determine the prevalence for NIHL and tinnitus, as well as attitude towards loud music and the factors influencing the use of protection devices. The 145 university students completed a questionnaire, with 89.5% experiencing transient tinnitus after loud music exposure, while 14.8% had permanent tinnitus, and only 11.0% using protection devices. A South African study conducted by Almec (2015) explored the risk perceptions of young people to music concerts and festivals, with a quantitative, descriptive questionnaire survey being conducted at five music venues to determine risk perception. Those who were less aware included 18-year-old – 20-year-old people, with most not using earplugs, and only one-third indicating that they would wear them if was enforceable by law.

As seen above, NIHL due to recreational noise is a serious health risk and concern. While many studies were conducted internationally, locally there is a paucity of data on NIHL among young South Africans, their understanding of noise-associated risks and what can be done to prevent and mitigate the effects. As young South Africans have varying access to noisy recreational activities and information about its consequences, it would be useful to establish the young adults’ awareness, attitudes and perceptions of leisure noise. Noise exposure in young adults can have serious long-term effects (Tambs, 2004). Therefore, it is important to establish what young people know and perceive about high noise levels. This is useful to create awareness, implement effective regulations and provide public education to young people. Such programmes will improve hearing health care of young adults. Arguably, awareness, attitudes and perceptions have a direct impact on quality of life and future employment, as noise can result in psychological and audiological effects on young adults (Tambs, 2004). The importance of education and health during the young adult phase is vital for a successful and positive future.

This study was conducted at a university in Durban and focused on students from the Education Department, who will one day be teachers and could therefore provide education regarding correct hearing health to their students. As there has been a paucity of research, this study aimed to determine the awareness, attitudes and perceptions of young adults towards leisure noise at a university in Durban, KZN, SA.

### Theoretical framework

This study was based on two theoretical frameworks, namely the Health Belief Model (HBM) (Hochbaum et al., 1952), and the Theory of Reasoned Action (TRA) (Fishbein, 1979), as they highlight the factors that influence awareness, attitudes and perceptions. The HBM highlights the function of beliefs in decision making and is often used to predict healthy behaviour (Abraham & Sheeran, 2015). The model suggests that people decide whether or not to change their behaviour based on the feasibility and benefits rather than the disadvantages and costs associated with a particular course of action (Naidoo & Wills, 2000). The model contends that a person is more likely to take preventative action against health issues if they feel a threat or risk to their health, regard themselves as susceptible to the threat, and see more benefits than costs by engaging in this action (Laranjo, 2016). It suggests that for a change of behaviour to occur, a person must have an incentive to change, feel threatened by the risks of their current behaviour, believe that the change will be beneficial, and be competent to carry out the change. People are therefore likely to change when they understand the long-term implications and effects, and why they should change (Naidoo & Wills, 2000). The HBM is based on six factors (Hochbaum et al., 1952), with an additional seventh modifying factor included by Offei (2017) which was also addressed in this study. The seven factors are: (1) Perceived susceptibility; (2) Perceived severity; (3) Perceived benefits; (4) Perceived barriers; (5) Cues (triggers) to action; (6) Self-efficacy; and (7) Demographics.

The second theory, the TRA, highlights the belief that behaviour is dependent on two variables: attitude and subjective norms. Attitudes refer to beliefs about the consequences of the behaviour and the implications of making a change (Naidoo & Wills, 2000), while subjective norms are what others do and expect you to do. These two variables combine to form an action, with the theory placing importance on social norms that make the role of modelling important (Naidoo & Wills, 2000). The theory states that attitude and norms lead to an intention or motivation and the possibility of a change in behaviour. People who have an intention to perform a behaviour or action will only carry it out if they evaluate it positively and believe that others will approve of them performing this behaviour (Mimiaga et al., 2009).

For the purpose of this study, the frameworks were used simultaneously (Conner & Norman, 2021). The HBM regarding beliefs and knowledge to be the leading factor to affect behaviours, while the TRA highlights the importance of attitudes and societal norms. All of the aspects are directly relevant for the study, as this study collectively encompasses awareness, attitudes and perceptions.

### Problem statement, aim and objective

There is a paucity of research in SA’s young adult population to determine their awareness, attitudes and perceptions towards leisure noise. This has implications as hearing loss can negatively impact health and well-being. As many young adults are not aware that they have a hearing loss, data on the extent of the problem in the country are limited. Young adults are highly influential as well as the future leaders and parents (Committee on Improving the Health et al., 2015), and are also the future contributors to the economy. Young adulthood is a critical age for the development of health and well-being. Correct awareness and hearing behaviour for young adults is vital. Conducting research in an academic institution that many young people attend, allowed the researcher an opportunity to access this population. Such evidence-based research related to leisure noise exposure could contribute to the hearing healthcare sector to determine the extent of the need for education and hearing conservation programmes. Such research could contribute to understanding what aspects to include in the education of the young adults based on results and frequency of use and attendance of leisure noise activities.

Therefore, the *aim* of the study was to determine the awareness, attitudes and perceptions of young adults towards leisure noise at a university in Durban, SA.

### Objectives

- To determine the young adults’ *awareness* about the risks of hearing loss and leisure noise.
- To determine the young adults’ *preferences* with regards to types of leisure noise and estimated intensity of the noise.
- To determine the young adults’ *attitudes and perceptions* about the effects of noise and appropriate preventive measures to help determine one’s willingness to change.
- To determine if there is a *relationship between* the level of awareness and attitudes regarding leisure noise.

## Research methods and design

### Study design, setting and population

A descriptive cross-sectional quantitative survey design, with an online self-administered questionnaire was considered in this study. Four hundred and sixty-two (462) registered undergraduate male and female students participated in this study. To be included, participants had to be 18 years old – 25 years old, have a Gmail account, and be in the Education Department of a university in Durban in the eThekwini district. Students who were excluded had a family history of hearing loss, were outside the age range and registered for post-graduate studies in the Education Department. The Department of Education was chosen as they are future educators and leaders whose general knowledge and attitudes are likely to play a part in their role of educating future generations (Kane & Francis, 2013).

### Sampling

Non-probability, purposive sampling was used to ensure selection based on the characteristics of a population. The university’s estimated population of 20 000 students with a confidence level of 95%, a 5% margin error and a response distribution of 50% resulted in the required sample size being a minimum of 377 students (Getahun, pers. comm., 24 April 2020). Participants were recruited via SMS sent by the departmental administrator, asking students to complete the survey via a link.

### Data collection

The self-administered, online survey consisted of closed-ended questions that was conducted via Google Forms, taking approximately 10 min to complete. The survey combined questions from three survey tools that directly aligned with the objectives of this study: WHO survey on ‘safe listening in entertainment venues’, with permission being requested but no response received (WHO, 2020); the publicly available survey on ‘Knowledge, behaviours and attitudes about hearing loss and hearing protection among ethnically diverse young adults’ from Chung et al. (2005), with no response being received. Permission from Crandell et al. (2004) to use the survey on the ‘Evaluation of NIHL in young people using a web-based survey technique’ was received.

The 32 questions required the respondent to select from various options, multiple-choice, rating scales and 3- and 5-point Likert and linear scale options. A pilot study was conducted with postgraduate students in the Education Department to determine the time required to complete the survey, its feasibility, costs as well as errors or insensitivity of the data collection tool. Minor grammatical changes were made from results of the pilot study.

The recruitment process and survey were active for 10 days on Google Forms. The study received 596 responses whereof 515 students agreed, with informed consent, to participate in the study. Of the 515 respondents who provided consent, 53 were excluded as they did not meet the inclusion criteria, which resulted in 462 viable responses, this being above the required 375 participants. Educational materials regarding leisure noise were also provided (in English and isiZulu) once the survey was completed.

### Data analysis

The data were first analysed descriptively to obtain the percentage of responses for the 32 questions, after which inferential analysis was done to address Objective 4. A chi-square test was used to determine the relationship between two categorical variables, and various categorical relationships were analysed by means of a *p* value of < 0.05 indicating a significant relationship (Statistics How To, 2024). Cronbach’s alpha (0.716) was used to analyse internal consistency, and a regression analysis was used to determine overall awareness of participants (Heale & Twycross, 2015).

### Ethical considerations

Ethical clearance to conduct this study was obtained from the Human and Social Sciences Research Ethics Committee of the University of KwaZulu-Natal (No. HSSREC/00002397/2021), and permission was obtained to conduct the survey with the School of Education undergraduate students. Permission was sought from the WHO, Crandell et al. (2004) and Chung et al. (2005) to use their surveys. The students were required to consent to participate on Google Forms before they could access the questionnaire. This process did not require students to divulge their names or personal information.

## Results

Out of 596 responses, 462 were eligible for the study. From the eligible participants, 318 (68.8%) were female, 208 (45%) were 18 years old – 20 years old, 288 (62%) were in their first or second year, and 385 (83.9%) were black. These statistics were reflective of the socio-demographic profile of university students in KwaZulu-Natal (University of KwaZulu-Natal, 2017).

### Objective 1: To determine the young adults’ awareness of hearing loss and the risks of leisure noise

Over half (59.2%, *n* = 273) had heard about hearing loss, and 84.6% (*n* = 390) knew that it could be caused by excessive noise, with 46.6% (*n* = 215) indicating that they are exposed to high levels of noise often. Almost half (48.3%, *n* = 223) were unsure whether sounds of over 80 dB can damage hearing permanently, while 47% (*n* = 217) were aware that it does (Table 1).

**TABLE 1:** Awareness about hearing loss and the risks of leisure noise (*N* = 461).

Level of awareness	*n*	%
**Does listening to noise at excessive levels cause a hearing loss?**		
No	71	15.4
Yes	390	84.6
**Do you feel that you are often exposed to very high levels of noise?**		
No	246	53.4
Yes	215	46.6
**Listening to sound over 80 decibels for an extended period of time can damage your hearing permanently.**		
False	22	4.8
True	217	47.0
I do not know	223	48.3
**Can hearing loss caused by noise be cured?**		
No	202	44.6
Yes	251	55.4
**How concerned are you about getting a hearing loss?**		
A very big concern	228	49.6
No concern	47	10.2
Not too big of a concern	90	19.6
Somewhat a concern	95	20.7

### Objective 2: To determine their preferences regarding types of leisure noise and estimated intensity of the noise

**FIGURE 1:**
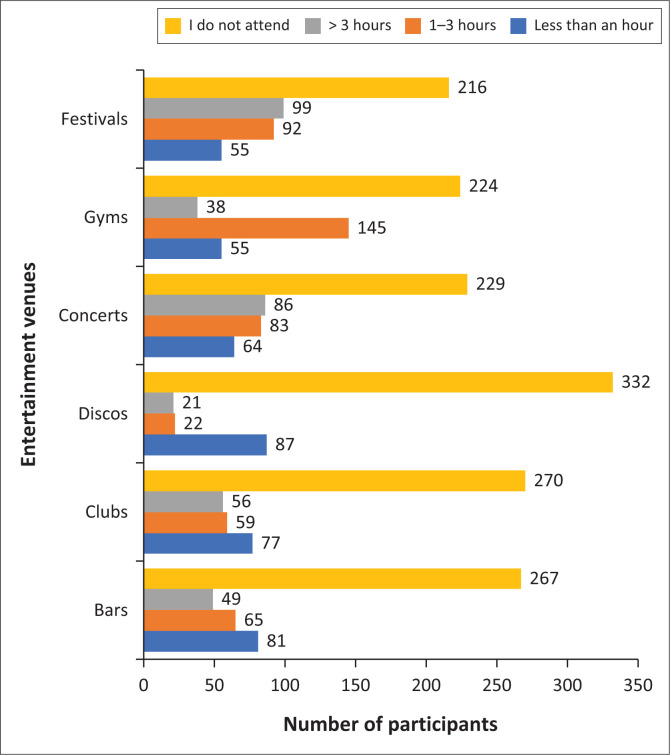
Duration of attendance at entertainment venues (*N* = 462).

Frequency of attendance of venues attended in descending order was restaurants (*n* = 241), gyms (*n* = 115), bars and clubs (*n* = 103), concerts (*n* = 92) and discos (*n* = 11). Time spent at particular venues varies, as indicated in Figure 1, with the sound ratings of the various venues in Table 2. A large number of participants did not attend the different venues, with only a few spending more than 3 h at each of these venues.

**TABLE 2:** Sound ratings of the various venues attended (*N* = 462).

Entertainment venues	Sound levels											
	Too high		High		Average		Low		Too low		Do not use or attend	
	*n*	%	*n*	%	*n*	%	*n*	%	*n*	%	*n*	%
Concerts/festivals	142	30.7	154	33.3	84	18.3	8	1.7	2	0.4	72	15.6
Discos	133	28.8	139	30.1	61	13.3	8	1.7	2	0.4	119	25.7
Personal devices	70	15.2	111	24.0	200	43.3	38	8.2	7	1.5	36	7.8
Gyms	24	5.3	78	16.9	217	46.9	57	12.3	12	2.6	74	16.0
Restaurants	13	2.8	38	8.2	248	53.7	107	23.2	16	3.5	40	8.6
Total†	382	16.5	520	22.5	810	35.0	218	9.4	39	1.7	341	14.7

† , *n* = 2310.

Participants were also asked to rate their perceived sound levels for bars and clubs, with almost half (48%, *n* = 211) finding the levels too high, while 15.9% (*n* = 70) found it too low. The majority (95.2%, *n* = 436) indicated that they use PLD, 29.4% (*n* = 134) used them at maximum volume, and 3.7% (*n* = 17) at barely audible volume, with 34.1% (*n* = 157) using it 1 h – 3 h a day, 36.9% (*N* = 170) less than an hour a day and 27.1% (*N* = 125) for more than 3 h a day. Earphones were the most preferred listening method (70.3%, *n* = 324), followed by over the ear earphones (22.85%, *n* = 105). Listening to music was the most common response for their uses (100%), followed by videos, phone calls, streaming and ‘other’.

Further analysis via inferential statistics (Table 3) indicated a significant association between time spent at a particular venue and the perceived sound levels, that is time spent at a disco versus the rate of sound (*p* = 0.002); concerts (*p* = 0.029); gyms (*p* = 0.000), with a negative correlation indicating that the more time the participant spent at the venue, the lower the sound was rated. A significant chi-square association was also found between time spent at gyms and the rating of sound of PLDs (*p* = 0.046) with a negative correlation.

**TABLE 3:** Time versus sound ratio.

Associations	Chi-square
How much of time do you usually spend at discos versus rate of sound at discos?	0.002*
How much of time do you usually spend at concerts versus rate of sound at concerts?	0.029*
How much of time do you usually spend at gyms versus rate of sound at gyms?	0.000*
How much of time do you usually spend at gyms versus rate of sound of personal listening devices?	0.046*

* , significant association.

According to the chi-square analysis, the use of PLDs and its loudness is closely related to the frequency of use of PLDs. As the perceived loudness of PLDs increased, so too did the prevalence of use of the device, indicating a significant relationship (*p* = 0.000). There was no significant association between the frequency of use and other variables, such as age (*p* = 0.511) and gender (*p* = 0.392).

### Objective 3: To determine their attitudes and perceptions towards preventive measures and the effects of noise to establish their willingness to change

Three quarters (69.5%, *n* = 321) experienced a problem after exposure to loud music, 16% (*n* = 74) after the gym, 34.2% (*n* = 158) after a club or restaurant and 38.7% (*n* = 179) after a concert. In addition, 214 indicated that they experience ringing in the ears most commonly, 166 had dizziness, 162 had ear pain, 128 had ear infections and 99 had trouble hearing after exposure to loud music. Furthermore, 68% (*n* = 313) stated that they experience ear pain, tinnitus or difficulty hearing, 15.2% (*n* = 59) that this occurs often and 32% (*n* = 147) that this does not occur.

**FIGURE 2:**
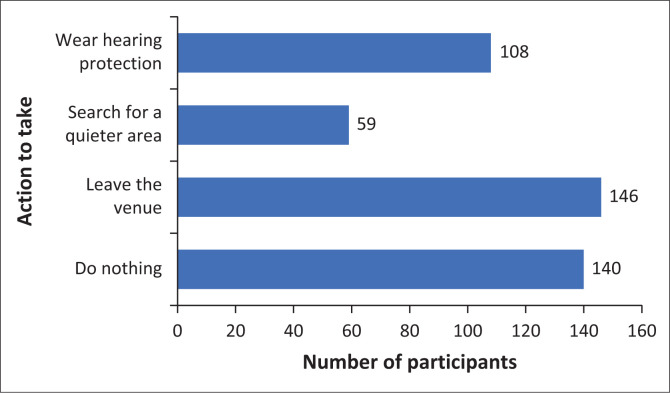
Most likely action in areas of high noise (*N* = 453).

Figure 2 indicates that 30.4% (*n* = 140) would be most likely to do nothing when in an environment with high noise levels, with many (72.1%, *n* = 328) acknowledging that hearing loss would be extremely disruptive for their lives. Knowledge about the use of earplugs was low, with only 10.4% (*n* = 48) having worn them previously. The majority (97.2%, *n* = 446) said they would decrease noise exposure and wear earplugs if they were aware of the risks of excessive noise. There were various reasons as to why they would or not wear earplugs, with 86.5% (*n* = 391) stating that if they were given them free in noise zones, they would wear them.

**FIGURE 3:**
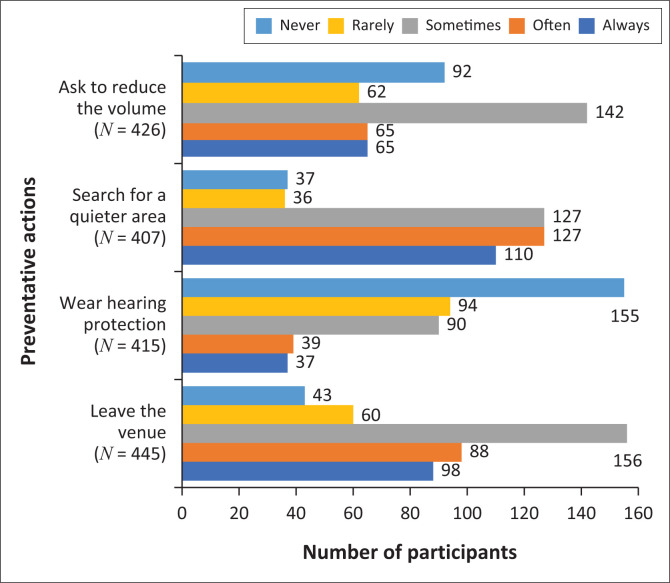
Action taken in loud areas.

Participants would rather leave the venue (22%, *n* = 98) or search for a quieter area (25.2%, *n* = 110) than ask for the noise to be reduced (15.3%, *n* = 65) or wear hearing protection (8.9%, *n* = 37) in a very noisy environment as seen in Figure 3. They indicate that they were only completely favourable of certain actions that included distributing earplugs (14.7%, *n* = 63), finding quiet zones (20%, *n* = 87), having volume limits (13%, *n* = 55), providing informational material (14.2%, *n* = 60) and posting warning signs (18.5%, *n* = 80).

### Objective 4: To determine if there is a relationship between the level of awareness and attitudes regarding leisure noise

Responses regarding awareness were combined and analysed in relation to each attitude question to establish any significant relationship between their awareness and attitudes. The chi-square analysis indicated an association (*p* = 0.029) between the level of awareness and most likely action in areas of loud noise, indicating that the higher the level of awareness, the more likely would the person act appropriately. The findings also indicated a significant relationship between the level of awareness and the perceived level of disruption a hearing loss would cause in their lives (*p* = 0.048) (Table 4). The better the overall awareness, the more likely they were to take action and to understand the consequences of hearing loss on their daily life.

**TABLE 4:** Chi-square analysis.

Relationship between awareness and attitudes	Chi-square
Level of awareness versus most likely action in loud noise	0.029*
Level of awareness versus perceived disruption in life	0.048*
Level of awareness versus favourability towards warning signs	0.047*

* , significant association.

## Discussion

### Objective 1: To determine the young adults’ awareness about hearing loss and the risks of leisure noise

The results indicate that despite an awareness of hearing loss generally, the specifics of noise and its effects on hearing loss were unknown. It showed that more than half (59.2%) of the participants had previously heard about hearing loss. More than half (55.4%) of the participants erroneously felt that hearing loss can be cured.

In contrast to this, Crandell et al. (2004) indicated that the majority of participants had a high level of knowledge regarding NIHL and its effects, with many (85%) knowing that hearing loss from noise cannot be cured. Gopal et al. (2019) reported that the majority of their participants (75%) were aware of NIHL. These differences could be attributed to these two studies being conducted in a developed country, whereas this study was in a developing country. This could be due to the more pressing burden of disease in SA as opposed to hearing being prioritised.

Chung et al. (2005) indicated an extremely low level of awareness, with only 16% of participants having read or heard anything related to hearing loss. This, in conjunction with the current study, emphasises the urgent need for hearing education at schools and the importance of teachers being educated. In another South African study, Seedat et al. (2020) reported that 30% of participants were unaware that listening devices increased the risk of a hearing loss. This is similar to the current study, suggesting that the results are consistent with other populations.

### Objective 2: To determine the young adults’ preferences with regards to types of leisure noise and estimated intensity of the noise

Approximately half (48%) of the participants found the noise levels of bars and clubs to be too high, this being lower than that of Beach and Gilliver (2019), who reported that 75% preferred sound levels below that played at these venues, and experienced hearing difficulties shortly after exposure to the sound. The implication is that not only is educating individuals on hearing health care important, but that venue owners need to consider their visitors’ noise level preferences (Beach & Gilliver, 2019).

Hussain et al. (2018), Govindsamy et al. (2020) and Seedat et al. (2020) all confirmed the high use of PLDs among young people. Similarly, a study conducted in Zimbabwe revealed that young people use earphones without knowing the risk of possible damage to hearing (Mutswanga & Makoni, 2014). The high use of PLDs in this study (95.2%) may be due to them being university students and expected to have access to a device to complete their work, specifically during the Coronavirus (COVID-19) pandemic, with the introduction of online and distant learning. The low attendance and duration of time at entertainment venues, are also limited due to the study being conducted during the pandemic and government restrictions.

In addition, 29.4% of participants use the PLDs at maximum volume, with 34.0% using it for more than 3 h a day, with most listening to music. Govindsamy et al. (2020) and Seedat et al. (2020) also noted the extensive use of PLDs, and that their volumes were increased in noisy environments, with most being unaware of the associated risks. Additionally, in this study, a significant chi-square association was also found between time spent at gyms and the rating of sound of PLDs (*p* = 0.046) while exercising, with a negative correlation. Research has indicated that high noise level in the gyms often results in people using their PLDs at even higher levels (Shuster & Hertzano, 2021), which has long-term detrimental effect to hearing.

While the current study indicated that 34.1% of the participants used earphones 1 h – 3 h a day, Tung and Chao (2013) reported that 90.9% of their participants regularly used them for an average of 1.6 h a day. These authors found that 29.9% stated that they sometimes require repetition while 22.9% often cannot hear people speak in noisy environments, with testing revealing that 11.9% already had hearing problems. The results of the current study indicated that only 16% of participants had read or heard anything related to hearing loss, with only 9% having heard about hearing loss at school, which also emphasises the urgent need for education on correct hearing health at academic institutions.

Hearing is essential for communication and education for students, with a failure to hear possibly resulting in feelings of depression and isolation (Themann et al., 2013). The current study reached the conclusion that the age group included has inadequate awareness regarding leisure noise, making education essential. As seen from the current study findings, as well as all other literature, PLD use is very high, with poor knowledge on risks of loudness and the effects of noise and hearing loss. The effects of hearing loss also seem to emerge in the young adults, who are at risk for increased hearing thresholds as well as tinnitus. This finding emphasises the need for increased awareness through education to encourage safe listening.

### Objective 3: To determine the young adults’ attitudes and perceptions towards preventive measures and the effects of noise to determine their willingness to change

The participants indicated that they experienced side effects after exposure to noise. In addition, 72.1% acknowledged that hearing loss would be extremely disruptive in their life. Knowledge about the use of earplugs was extremely low, with 90% having never worn them, which indicates the low level of awareness among the age group. This could indicate that even if awareness exists, participants were unlikely to take action to decrease the risk of hearing loss. However, 97.2% of participants said that they would decrease noise exposure and wear earplugs if they were aware of the risks of excessive noise, indicating the importance of education to influence behaviour change.

The majority (86.5%) of participants stated that if earplugs were given to them for free in noise zones, they would be willing to wear them. This shows the importance of establishing policies, legislation and regulation (WHO, 2019b) in SA, similar to those in Switzerland, for individuals to be protected from developing a hearing loss due to leisure noise exposure. It also highlights the importance of venues being informed and educated about hearing health in order to protect all venue attendees. This result concurs with the TRA, which emphasises the importance of social norms and attitudes.

World Health Organization and Kamenov (2019) indicated that 39% of participants wore protection devices, this being in contrast to the current study, with only 10.4% having worn them previously. Both results are possibly being due to the lack of education regarding the effects of high noise levels. Chung et al. (2005) reported that 61% of young adults experience tinnitus and 43% experience TTS after exposure to loud music, which is directly comparable to the current study. They found that only 14% of participants reported wearing Hearing Protection Devices (HPDs) in areas of loud noise, which concurs with the current study results and indicates the high occurrence of symptoms post noise exposure, as well as an extremely low percentage of HPD use.

Holmes et al. (2007) also found that many people experienced TTS and pain with loud noise exposure as well as perceived tinnitus and hearing loss, but that the use of HPDs was limited. Permanent threshold shift was experienced by 14.8% of their participants, while only 11.0% used protection device, similar to the current study. This highlights that the lack of knowledge in adults and the non-usage of protection devices should be targeted for preventative measures (Gilles et al., 2012).

### Objective 4: To determine if there is a relationship between the level of awareness and attitudes regarding leisure noise

The responses were combined and analysed to determine if there was a significant relationship between the participants’ level of awareness of the effect of noise and attitudes. According to the chi-square analysis, a significant relationship (*p* = 0.029) was found between the level of awareness compared to the participants’ most likely action in areas of loud noise. This indicates that the higher the level of awareness, the more likely participants were to act appropriately. There was a significant relationship between the level of awareness of participants and the perceived level of disruption a hearing loss would cause in their lives (*p* = 0.048). This confirms that the better the overall awareness, the more aware participants are on the effect of hearing loss on their daily life. These two results suggest that awareness and behaviour as well as perceptions are directly linked, that the greater the awareness, the less likely the risk of voluntary high noise exposure and hearing loss due to avoidance behaviour.

The study results provide clear evidence of a lack of awareness in young adults towards leisure noise in particular, and that attitudes and perceptions influence behaviour. Collectively, the study indicated the urgent need for education within this study population to not only influence their individual health behaviour change, but to influence education and health promotion in their future jobs, by educating the youth of today, the future of tomorrow.

### Relevance of the frameworks

As guided by the HBM, the study results indicated that the participants were more likely to have better attitudes and health behaviours if they were aware of the risks associated with loud noise on their hearing and highlights the importance of correct education. Participants did not regard their hearing health as a priority, hence the limited use of HPDs and the acceptance of loud noises in entertainment venues. This is in accordance with the HBM, which contends that behaviour change only occurs in relation to the susceptibility to and severity of the likely consequences, and the perceived benefits.

Social norms and attitudes proved to be a major factor of the current study and highlight the importance of correct education and knowledge. This theory also emphasises the need for laws and regulations regarding noise levels in entertainment venues and the use of HPDs. The theory has implications for clinical practices as it informs audiologists about factors that will assist young adults to be more likely to change their behaviour.

### Limitations

A number of limitations are acknowledged, including that it only focused on students studying for a degree in education at one university, limiting their generalisability to other university students. The study was quantitative in nature and did not provide the respondents with an opportunity to express any in-depth opinions. There was an unequal gender and racial distribution. The study was also limited due to the COVID-19 pandemic restricting the frequency of attending entertainment venues. Lastly, no hearing thresholds were obtained from the participants.

### Clinical implications of the study

Implications of the study include influencing audiologists’ education of young adults. This is as it provides information on the extent of education required and the need for hearing conservation programmes, helps indicate what factors regarding awareness are lacking in an education context, how to resolve this issue within the teaching curriculum, as well as informs audiologists how best to impact the populations’ attitudes and perceptions towards noise and correct hearing health. It will also enable the research results to be applied in the larger population, as the results will allow universities to conduct wide-scale education programmes that advocate for good hearing health in all sectors. This can be achieved via part of a mandatory community health module for all students as well as hearing screening programmes. The Department of Basic Education needs to include hearing health education as part of the school curriculum. The findings of this study can be shared with the Department of Basic Education and universities to influence change in practice in education regarding hearing health and the effects of noise.

### Recommendations for future research

Recommendations from this study include further research in the following areas:

- Similar topics, including threshold testing, to establish the likelihood of NIHL among young adults.
- Include the entire university student body for more large-scale results to allow for generalisation across a broader context.
- Focus more on educating young adults on noise and its risks as opposed to only determining whether education is warranted.
- Conduct a study of similar nature on young adults in general, and not specifically university students to allow for generalisation.
- Repeat a study of a similar nature, post the COVID-19 pandemic. This is to determine the impact of frequency of attendance to venues or other aspects when there are no government regulations.

## Conclusion

The results from this study indicated that the participants had some awareness regarding hearing loss in general, but lacked specific knowledge on hearing loss, noise, and its effects. The results revealed that PLDs are a major aspect of young adults’ life. More than half (69.5%) of participants said that they had experienced some side effects such as tinnitus and dizziness after exposure to noise. It was also evident that majority of participants were not aware of hearing protection devices. The results indicated that as participants awareness increased, their attitude was likely to improve regarding, thus reducing their risk to a NIHL.

Collectively, the study revealed that attitudes are linked to awareness, which directly influences behaviour, and that greater awareness is required through hearing conservation programmes and education to advocate for better hearing health and correct listening in noise habits. The results indicate that education and training is vital. The study highlights the urgent need for action with regards to improving the general awareness and hearing health of the university students and young adults in general. There is a considerable demand for all relevant stakeholders to increase awareness on hearing health care education within the South African context. This can be done via appropriate education and health promotion to the public as recommended by the WHO (2019). The findings also determine the need for a hearing conservation programme that involves leisure noise activities and recommended means of protecting hearing. This will allow young adults to better manage their risk of hearing loss via safe listening measures, as indicated by the study’s theoretical framework. This would also ensure a better quality of life and future employment opportunities for young adults (Tambs, 2004). There is also an urgent need for student teachers to be educated and to educate learners about the risks associated with high noise volumes. In addition, the South African government needs to prioritise and urgently develop adequate laws and guidelines for safe listening habits in entertainment venues.
